# Economic Evaluation of Adding Bevacizumab to Chemotherapy for Metastatic Colorectal Cancer (mCRC) Patients in Indonesia

**DOI:** 10.31557/APJCP.2021.22.6.1921

**Published:** 2021-06

**Authors:** Erna Kristin, Dwi Endarti, Levina Chandra Khoe, Kartika Widayati Taroeno-Hariadi, Christina Trijayanti, Armansyah Armansyah, Sudigdo Sastroasmoro

**Affiliations:** 1 *Department of Pharmacology and Therapy, Faculty of Medicine Public Health and Nursing, Gadjah Mada University, Yogyakarta, Indonesia. *; 2 *Department of Pharmaceutics, Faculty of Pharmacy, Gadjah Mada University, Yogyakarta, Indonesia. *; 3 *Department of Community Medicine, Faculty of Medicine, Universitas Indonesia, Jakarta, Indonesia.*; 4 *Respira Hospital, Yogyakarta, Indonesia. *; 5 *Center of Financing and Health Insurance, Ministry of Health, Government of Indonesia, Indonesia. *; 6 *Department of Pediatrics, Faculty of Medicine, Universitas Indonesia, Jakarta, Indonesia. *

**Keywords:** Metastatic colorectal cancer, bevacizumab, chemotherapy

## Abstract

**Objective:**

Since 2016, bevacizumab has been widely used to treat metastatic colorectal cancer (mCRC) in Indonesia. Nevertheless, the high cost of bevacizumab has raised the question of whether the therapy is considered cost-effective and should be included in the national health insurance system. This study aimed to assess the cost-effectiveness of bevacizumab plus chemotherapy versus chemotherapy alone for the treatment of mCRC patients.

**Methods:**

A Markov model was applied using the perspective of the Indonesian healthcare system to assess cost-effectiveness. The health outcomes were expressed in terms of quality-adjusted life years (QALY) using the validated EuroQoL-5D-5L instrument. Data for medical costs were collected from hospital billings in four hospitals located in three different cities in Indonesia. Meanwhile, data for utility were obtained from interviewing 90 patients who came to the hospital. We compared those mCRC patients who received chemotherapy alone either with FOLFOX or FOLFIRI, versus patients who received the addition of bevacizumab.

**Results:**

With the perspective of societal, the incremental cost-effectiveness ratio (ICER) of adding bevacizumab was USD 49,312 per QALY gained using secondary data and USD 28,446 per QALY using real world data.

**Conclusion:**

Using either a healthcare or societal perspective, the addition of bevacizumab for mCRC treatment was considered not cost-effective.

## Introduction

Colorectal cancer ranks as the third most common cancer worldwide, with nearly 750,000 new cases (10% of cancer cases) and more than 370,000 deaths (8% of all cancer deaths) per year. The highest incidence and number of deaths are found in Southeast Asia, with 68,000 new cases and 48,000 deaths (Ferlay et al., 2013). In Indonesia, colorectal cancer has entered in the top 10 most common cancers. An incidence rate of 12.1 per 100,000 adults was reported in 2018 (GLOBOCAN, 2018). 

For metastatic colorectal cancer (mCRC) patients, the standard chemotherapy regimens are 5-fluorouracil (5-FU), leucovorin, and oxaliplatin (FOLFOX), or oxaliplatin with capecitabine (CAPOX), or 5-FU, leucovodin, and irinotecan (FOLFIRI). Targeted therapies (i.e., bevacizumab, cetuximab, and panitumumab) can be added into these standard regimens (Ministry of Health, 2015). Bevacizumab (Avastin) is a recombinant humanized monoclonal antibody targeting the vascular endothelial growth factor (VEGF). VEGF is a mediator of tumor angiogenesis. Bevacizumab blocks the binding of VEGF to its receptor, thus inhibiting tumor growth. Botrel et al (2016) reported the clinical effectiveness of bevacizumab and found a better response rate and increased survival rate compared with chemotherapy alone. Another systematic review conducted by Llic et al., (2016) revealed similar results. The overall survival and progression-free survival were better in the group with combination chemotherapy and bevacizumab compared with the chemotherapy group, i.e. HR: 0.84; 95% CI: 0.74-0.94; p < 0.05 and HR: 0.64; 95% CI: 0.55-0.73; p< 0.05, respectively (Llic et al., 2016). 

The addition of bevacizumab into mCRC chemotherapy regimens was introduced in Indonesia in 2006. The National Agency of Drug and Food Control (BPOM) has approved the use of bevacizumab for mCRC in combination with FOLFOX or FOLFIRI. Nevertheless, there is still controversy regarding the high cost of bevacizumab. It was estimated that one mCRC patient needed at least 57 million IDR (i.e., USD 4000) for four cycles. Therefore, this study aimed to assess the cost-utility of bevacizumab as an adjuvant therapy for mCRC patients. 

## Materials and Methods

*Data Collection*

An economic evaluation was conducted to determine the cost-utility of currently available bevacizumab in addition to standard chemotherapy regimen versus chemotherapy alone in managing mCRC patients under national health insurance. The study population was mCRC patients who received chemotherapy (i.e., FOLFOX or FOLFIRI) alone and those who received additional bevacizumab on top of their standard chemotherapy. Data were obtained from four hospitals located in three different cities in Indonesia: Jakarta, Yogyakarta, and Bali. Direct medical costs were taken from hospital billings. Indirect costs and utility data were asked directly from patients using validated questionnaires. We analyzed the data using both a healthcare perspective and societal perspective.

*Data Analysis*

The analysis was performed using a three-state Markov model built in Microsoft Excel, which was adopted from previous studies (Sherman et al., 2019; Ungari et al., 2017). It was designed to encapsulate the transition of mCRC patients through the following health states: progression-free, progressive disease, and death. The transition probabilities were obtained from systematic review, which were then confirmed with real world data. A two-month cycle with a lifetime horizon and 3% discount rate were applied in this model to capture all relevant costs and health benefits. The time horizon applied in the model starting at age 50 corresponded to the mean age of the trial population at diagnosis. 

*Clinical Outcomes*

We used two approaches to obtain the clinical outcomes: a systematic review and survival analysis using real world data. The systematic review was performed on PubMed, Cochrane, Clinical Evidence, EMBASE, and CINAHL, using the terms “Bevacizumab”, “overall survival”, “progression-free survival”, and “metastatic colorectal”. The inclusion criteria were a systematic review or randomized controlled trial on mCRC patients, receiving a combination of chemotherapy that included bevacizumab, adult population, resectable tumor, and patients diagnosed with de novo metastatic colorectal cancer. Exclusion criteria were those that included metastases to brain. Articles were limited to full-text English literature, with the publication year after 2000. The selected articles were assessed for methodological quality using the Jadad scale. We also conducted a cohort retrospective using medical records from 4 hospitals, with a minimum sample of 75 medical records in each health state.

*Costs*

The costs were calculated using a societal perspective. Data were obtained primarily from hospital billing and interviews with patients or caregivers. Direct medical costs included drug costs, hospitalization, consultation fees, laboratory and imaging costs, and hospital administration costs. We also interviewed patients to obtain indirect and direct non-medical costs. These included transportation costs, meal and lodging costs (if applicable) for patient and caregiver during control, and the cost of lost productivity due to illness.

*Utilities*

Health utilities were obtained directly from mCRC patients using a validated EuroQoL-5D-5L instrument. We interviewed 90 cancer patients with 15 patients per health state in each hospital. All respondents were not limited to mCRC patients due to the small number of mCRC patients. As such, we also included other patients with advanced cancer.

*Sensitivity Analysis*

Probabilistic sensitivity analyses were undertaken to address the uncertainty in the model assumptions in which all parameters were run simultaneously by 1,000 iterations. A cost-effectiveness acceptability analysis was conducted to compare the strategy between adding bevacizumab to chemotherapy versus chemotherapy alone at various willingness-to-pay thresholds. The WTP threshold was set as three times the GDP per capita in 2017, which was $10,800/QALY in Indonesia.

## Results

*Clinical Outcomes*

A total of eight randomized clinical trials were included in our review. From these trials, four of them measured overall survival. We also assessed the quality of these studies using the Jadad scale and found that the score ranged between 2 and 4. The one-year survival of mCRC patients with the addition of Bevacizumab compared to chemotherapy alone were generally better, i.e., IFL/placebo versus IFL/Bevacizumab (63.4% vs. 74.3%); 5-FU/LV/placebo versus 5-FU/LV/Bevacizumab (53% vs. 63%); XELOX/FOLFOX4/placebo versus XELOX/FOLFOX4/Bevacizumab (72% vs. 79%), FOLFOX4 versus FOLFOX4/Bevacizumab (43% vs. 56%). The range of median survival time in patients with chemotherapy alone was 10.8 to 19.9 months, while those with bevacizumab was 12.9 to 21.3 months (Kabbinavar et al., 2005; Stathopoulos et al., 2010). Real world data obtained from medical records were relatively different; the median survival of patients with bevacizumab and chemotherapy (n=96) was 12.5 months and those with chemotherapy only (n = 43) was 8.8 months.

*Patient’s Characteristics*

There were 139 patients included in this study with 96 of them receiving chemotherapy and bevacizumab. The number of males and females was pretty similar (51.8% vs. 48.2%). The mean age was 53.7±11.2 years old. The chemotherapy regimen commonly used among the patients were a combination of oxaliplatin plus capecitabine (43.9%), oxaliplatin plus leucovorin and 5-FU (42.5%), or irinotecan plus leucovorin and 5-FU (13.7%).

*Input Parameter*

The following parameters were used in our modeling: transition probabilities from a systematic review, transition probabilities from real world data, clinical effectiveness from a systematic review, costs (direct and indirect), and utilities. We ran the model using data from a systematic review and real-world data to see if there were any differences.

*Incremental Cost-Effectiveness Ratio (ICER)*

In the base case analysis, using the perspective of healthcare provider, the total cost for the chemotherapy regimen FOLFOX/FOLFIRI/XELOX was 359 million IDR per patient with a mean average of 1.90 QALY over a life-time horizon. Bevacizumab treatment was associated with an additional cost of 108 million IDR and an additional 0.17 QALY. Using secondary data, treatment with bevacizumab resulted in an ICER of 653 million IDR to gain one additional QALY. In the real-world data, the ICER was much lower, i.e., 354 million IDR per QALY gained. We also ran the analysis using a societal perspective, as described in Table 2.

*Sensitivity Analysis*

We also ran a probabilistic sensitivity analysis (PSA) using both secondary data and real-world data. A cost-effectiveness acceptability curve was plotted based on the PSA, which showed that the likelihood of adding bevacizumab into standard chemotherapy was considered cost-effective between a 40% and 60% willingness-to-pay threshold of 250 million IDR. This increased to nearly 100% at 300 million IDR. This was applied for secondary data. For real-world data, the addition of bevacizumab was considered 80% cost-effective at 300 million IDR. Both findings showed that if we used three times GDP as the threshold, bevacizumab was not a cost-effective treatment for mCRC.

**Table 1:** Input Parameters for the Markov Model

Input Parameter	Base-case value	SE
Transition probabilities (Tp) of chemotherapy alone using systematic review		
Tp progression-free to progression-free	0.877	α = 125; β = 15
Tp progression-free to progressive disease	0.116	-
Tp progression-free to death	0.006	-
Tp progressive to progressive disease	0.963	-
Tp progressive disease to death	0.037	α = 124; β = 33
Transition probabilities (Tp) of chemotherapy alone using real world data		
Tp progression-free to progression-free	0.950	-
Tp progression-free to progressive disease	0.043	α = 124; β = 33
Tp progression-free to death	0.006	-
Tp progressive to progressive disease	0.974	-
Tp progressive disease to death	0.026	α = 3; β = 13
Transition probabilities (Tp) of chemotherapy plus Bevacizumab using real world data		
Tp progression-free to progression-free	0.955	-
Tp progression-free to progressive disease	0.038	α = 35; β = 61
Tp progression-free to death	0.006	-
Tp progressive to progressive disease	0.957	-
Tp progressive disease to death	0.043	α = 7; β = 28
Risk ratio (chemotherapy + bevacizumab vs. chemotherapy) using systematic review		
Progression-free survival	0.720	0.0306
Overall survival	0.840	0.0357
Costs (in IDR)		
Direct medical cost patient in stable state (chemotherapy alone)	14,671,335	2,001,108
Direct medical cost patient in stable state (chemotherapy + bevacizumab)	16,581,540	1,297,381
Direct medical cost patient in progressive state (chemotherapy alone)	11,002,640	3,215,142
Direct medical cost patient in progressive state (chemotherapy + bevacizumab)	13,571,535	3,045,121
Direct non-medical cost patient in stable state (chemotherapy alone)	1,585,498	1,585,498
Direct non-medical cost patient in stable state (chemotherapy + bevacizumab)	1,635,927	1,635,927
Direct non-medical cost patient in progressive state (chemotherapy alone)	1,585,498	1,585,498
Direct non-medical cost patient in progressive state (chemotherapy + bevacizumab)	1,635,927	1,635,927
Indirect cost patient in stable state (chemotherapy alone)	225,150	225,150
Indirect cost patient in stable state (chemotherapy + bevacizumab)	137,468	137,468
Indirect cost patient in progressive state (chemotherapy alone)	225,150	225,150
Indirect cost patient in progressive state (chemotherapy + bevacizumab)	137,468	137,468
Utilities		
Progression-free state (chemotherapy alone)	0.864	0.070
Progressive state (chemotherapy alone)	0.724	0.070
Progression-free state (chemotherapy + bevacizumab)	0.793	0.107
Progressive state (chemotherapy + bevacizumab)	0.659	0.131
Discount rate		
Discount rate for costs	0.03	-
Discount rate for outcome	0.03	-

**Table 2:** Results of the Cost-Utility Analysis Using a Societal Perspective

Treatment	Secondary data			Real-world data		
	Cost (USD)	QALY	ICER per QALY	Cost (USD)	QALY	ICER per QALY
FOLFOX/FOLFIRI/XELOX	30,552	1.90	49,312	42,789	2.62	28,446
FOLFOX/FOLFIRI/XELOX + Bevacizumab	38,720	2.07		67,774	3.50	

**Figure 1:**
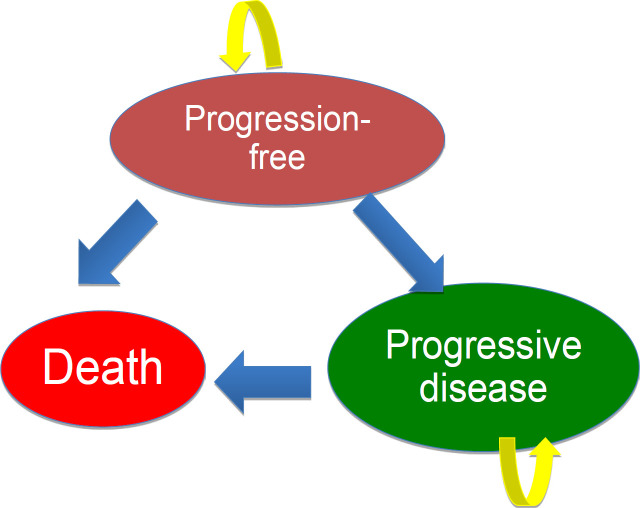
Three-State Markov Model

**Figure 2:**
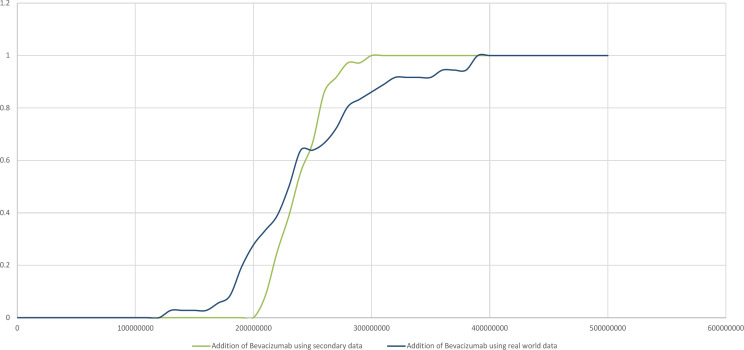
Cost-Effectiveness Acceptability Curve: chemotherapy plus bevacizumab versus chemotherapy alone

## Discussion

Across eight clinical trials, we found that the overall survival associated with the treatment of bevacizumab and chemotherapy was slightly better than that with chemotherapy alone. The results from real-world data in Indonesia, however, showed a lower median survival in groups treated with chemotherapy alone (8.8 vs. 12.9 months) and also with the addition of bevacizumab (12.5 vs. 21.3 months), compared with data from systematic review. Several factors could be linked with the difference of survival rate between overseas studies and local studies, such as combination chemotherapy (Llic et al., 2016), performance status (Llic et al., 2016), metastatic organ site (Wang et al., 2020), location of primary tumors (Loupakis et al., 2018), etc. In terms of combination chemotherapy, our data showed oxaliplatin plus capecitabine (XELOX) and oxaliplatin plus leucovorin and 5-FU (FOLFOX) as the most common combination chemotherapy used for mCRC patients. These were not much different than the chemotherapy regimen recorded in overseas studies. We should also be aware that the majority of patients presented to hospitals in Indonesia arrived in poor general condition. In addition, there were socioeconomic factors that likely affected patients’ compliance to chemotherapy procedures, i.e., chemotherapy frequency, hospital access, transportation cost, and caregiver’s time. Unfortunately, in this study, we did not assess the possible effect of these factors towards a patient’s survival outcomes. We compared both data from the systematic review and real-world data to validate the model. Data from the real-world showed a better survival rate in the long-term compared to the secondary data.

We also noticed that this Markov model merely examined the first-line therapy, and was not included in the patient’s movement from first-line to second-line therapy. We generalized the combination chemotherapy (XELOX, FOLFOX, and IFL) and did not conduct a separate sub-group analysis to observe the effect of these variation on clinical and economic outcomes.

In regard to cost-utility analysis, we noted the limitations associated with the methodology. The utility parameters were obtained from patients diagnosed with advanced cancer, and not specific to mCRC. Additionally, the costs were taken from hospital billings that might not represent the real expense from healthcare providers. Costs and utility data were also obtained from four hospitals, which might not be sufficient to capture the variation in Indonesia. Nevertheless, these were the best available evidence in our setting. Understanding the uncertainty in these parameters, we evaluated by running probabilistic sensitivity analysis with 1,000 simulations. Using the threshold of three GDP per capita, we found the ICER value were far above by using both secondary data and real-world data. These results aligned with other cost-effectiveness studies on bevacizumab that considered adding bevacizumab as an ineffective strategy (Sherman et al., 2019; Ungari et al., 2017).

Nevertheless, our findings make important contributions to decision-makers. First, the study presents healthcare costs related to mCRC treatment from the perspective of providers and patients. Direct non-medical costs and indirect costs were high in variation and our study was able to present this information. Second, it provides valuable evidence on the cost-effectiveness analysis that could be used to consider treatment strategies for mCRC patients under the national health insurance system, and whether bevacizumab should still be included in the benefit package.

In conclusion, the cost-utility analysis of bevacizumab plus chemotherapy versus chemotherapy alone as a first-line treatment for mCRC patients resulted in an ICER of USD 28,446 per QALY gained using real-world data and ICER of USD 49,312 per QALY gained using secondary data based on societal perspective. These ICER values were far above the threshold of three GDP per capita.

## Author Contribution Statement

The authors confirm contribution to the paper as follows: study conception and design: EK, DE, A, SS; data collection: KW, CT; analysis and interpretation of the results: EK, DE, LC, KW, CT; draft manuscript preparation: LC, EK, DE. All authors reviewed the results and approved the final version of the manuscript. 

## Approval by scientific Body

The study was assigned and approved by the Indonesian Health Technology Assessment Committee, the Center of Financing and Health Insurance, Ministry of Health, Government of Indonesia.
